# Cetrorelix promotes cell apoptosis via the PI3K–AKT–FOXO1 pathway in epithelial ovarian cancer

**DOI:** 10.3389/fonc.2025.1631576

**Published:** 2025-12-12

**Authors:** Ning Zhang, Yu Liu, Xiaodan Zhang, Meng Xie, Ying Zhang, Keqin Hua

**Affiliations:** 1 Department of Gynecology, Obstetrics and Gynecology Hospital, Fudan University, Shanghai, China; 2 Shanghai Key Lab of Reproduction and Development, Shanghai Key Lab of Female Reproductive Endocrine Related Diseases, Shanghai, China

**Keywords:** gonadotropin-releasing hormone antagonist, cetrorelix, apoptosis, epithelial ovarian cancer, PI3K–AKT–FOXO1 pathway

## Abstract

**Introduction:**

Epithelial ovarian cancer (EOC) has a dismal prognosis, and recent therapeutic advancements have been limited. The aim of our study was to clarify the role and mechanism of cetrorelix in EOC apoptosis and to evaluate the clinical relevance of GnRHR, AKT, and FOXO1 in EOC patients.

**Methods:**

Apoptosis was assessed using flow cytometry, Hoechst staining, and Western blotting. FOXO1, p-AKT and GnRHR knockdown via siRNA was performed to reverse cetrorelix-induced apoptosis. Mechanistic insights were explored using apoptosis gene PCR arrays, qRT–PCR, and Western blotting. *In vivo* efficacy was tested in a xenograft mouse model. Immunohistochemistry (IHC) was used to assess GnRHR, AKT,p-AKT and FOXO1 expression in EOC tissues, and survival analysis was performed using Kaplan–Meier and Cox regression analyses.

**Results:**

Cetrorelix facilitated EOC apoptosis both *in vitro* and in a xenograft model. The results of apoptosis PCR arrays linked cetrorelix treatment to the upregulation of the TNF/TNF receptor superfamily (a FOXO1-dependent mechanism). Mechanistically, cetrorelix upregulated FOXO1 expression, and FOXO1 knockdown attenuated cetrorelix-induced apoptosis. Furthermore, cetrorelix-mediated suppression of p-AKT expression and subsequent FOXO1 activation occurred via the PI3K/AKT signaling axis. This mechanism was substantiated by the findings that the PI3K inhibitor LY294002 mimicked cetrorelix’s effects without producing an additive apoptotic response, and that GnRHR knockdown abrogated cetrorelix-induced apoptosis, confirming receptor specificity. Experiments in xenograft models recapitulated the PI3K/AKT/FOXO1 cascade modulation observed *in vitro*. However, the *in vivo* activation status of FOXO1 was not quantitatively assessed or localized within the xenograft tissues. Clinically, FOXO1/GnRHR positivity and AKT negativity were correlated with early-stage disease (FIGO I-II, *p* < 0.05), no metastasis (*p* < 0.05), and improved survival (log-rank *p* < 0.05). Multivariate analysis revealed GnRHR positivity, AKT negativity, low-grade pathological type and early FIGO stage as independent risk factors for improved overall survival.

**Conclusion:**

These findings suggest that cetrorelix may induce EOC apoptosis via the PI3K/AKT–FOXO1 pathway, which provides mechanistic support for the therapeutic potential of GnRH antagonists in EOC management. Moreover, the identified critical regulatory pathways are prospective therapeutic targets for EOC management.

## 1 Introduction

Epithelial ovarian cancer (EOC) has a dismal prognosis characterized by a 5-year survival rate of 47% (1). The primary therapeutic strategy for epithelial ovarian cancer involves aggressive tumor debulking procedures accompanied by platinum-based and taxane compound chemotherapy. However, patients with advanced-stage tumors often have suboptimal treatment outcomes, and there have been no significant breakthroughs in improving the 5-year survival rate for ovarian cancer patients (2). To date, therapeutic progress for EOC has been slow, and the development of effective treatments for this disease is becoming increasingly urgent.

As the second leading cause of gynecological cancer-related mortality following uterine malignancies (3), EOC exhibits aggressive progression characteristics that correlate with the hormonally active microenvironment of the ovary (4). Emerging evidence from preclinical models and epidemiological studies substantiates the critical role of hormone signaling in EOC pathogenesis (5). According to the ‘gonadotropin theory’ (6), follicle-stimulating hormone (FSH) and luteinizing hormone (LH) promote ovarian cancer growth. The secretion of these hormones is mediated by gonadotropin-releasing hormone (GnRH). GnRH antagonists inhibit gonadotropin secretion by competitively binding to the GnRH receptor (GnRHR). GnRH antagonists have been used in clinical studies to treat hormone-dependent tumors, such as breast cancer (7), prostate cancer (8), and uterine fibrosis (9). Mechanistic studies have revealed that GnRH antagonists exert time- and dose-dependent growth suppression effects through GnRHR in multiple cancer types (10, 11). Antagonistic analogs of GnRH and GnRH-II directly induce endometrial and ovarian cancer cell apoptosis through a dose-dependent loss of mitochondrial membrane potential and subsequent activation of caspase-3 (12). The above evidence indicates that GnRH antagonists and GnRHR represent promising frontiers for the development of novel antitumor therapies in clinical oncology. Given its relatively low drug toxicity and potential adverse effects compared with those of chemotherapy, the specific anticancer effect of GnRH antagonists/GnRHR in ovarian cancer deserves further exploration.

Currently, the effects of GnRH antagonists on EOC cell apoptosis and the underlying molecular pathways have yet to be elucidated. We systematically investigated the effects of cetrorelix, a clinically available GnRH antagonist, and demonstrated its ability to induce EOC cell apoptosis through PI3K/AKT-mediated FOXO1 activation in both *in vitro* (cell-based) and *in vivo* (xenograft) models. Clinically, the low expression of GnRHR or FOXO1 and high expression of AKT correlate with tumor aggressiveness and unfavorable clinical outcomes, suggesting their utility as novel prognostic biomarkers in EOC patients. Our study enhances the understanding of the biological processes governing the impact of GnRH antagonists on EOC and provides research evidence for the application of GnRH antagonists as a promising therapeutic strategy for EOC. Furthermore, our study revealed critical components in regulatory pathways that may be molecular targets for developing precision therapies against EOC.

## 2 Materials and methods

### 2.1 Cell lines and reagents

The University of Texas MD Anderson Cancer Center (Houston, TX, USA) generously donated the human EOC cell lines (A2780, SKOV3 and SKOV3-ip). All the cells were cultivated in RPMI-1640 medium supplemented with 10% FBS and 1% penicillin/streptomycin (all from Gibco, Grand Island, NY, USA) in a 37 °C humidified environment with 5% CO_2_.

To produce a 100 mM stock solution, the GnRH antagonist cetrorelix (Sigma–Aldrich, St. Louis, MO, USA) was solubilized in 1 mol/L hydrochloric acid (HCl). Aliquots were diluted 100-fold, and the solution pH was 7.

### 2.2 Cetrorelix stimulation

EOC cells were cultured in 6-well plates for 24 hours. After reaching 60–70% confluency, the cells were subjected to serum deprivation (incubation in serum-free medium) for 18 hours. At the time of drug administration, the serum-free medium was replaced with fresh complete medium containing 10% FBS. For dose–response studies, cultures were exposed to cetrorelix at logarithmic concentrations (1 nM to 1 mM) over defined durations, and equivalent concentrations of HCl solution served as the vehicle control. Apoptotic profiling assays (flow cytometry) and molecular analyses (PCR arrays, qRT–PCR, immunoblotting) employed a fixed cetrorelix concentration of 100μM. Transfected cell cohorts followed identical serum starvation and medium replacement protocols prior to 48 hours of treatment with 100μM cetrorelix. After treatment, the cells were processed for apoptosis assessment via flow cytometry, whereas lysates were prepared for downstream protein or RNA quantification by Western blotting or quantitative reverse transcription PCR.

### 2.3 Apoptosis assay

Apoptosis was assessed using either a Hoechst staining kit (Beyotime Institute of Biotechnology, Shanghai, China) or a V-PE apoptosis detection kit (BD Franklin Lakes, NJ, USA) following the standardized protocols provided by the manufacturers. EOC cells were serum-starved for 18 hours to synchronize cell cycles. Prior to drug treatment, the serum-free medium was replaced with complete medium (10% FBS) containing cetrorelix or HCl control. Following the specified exposure durations, the cells were retrieved, washed twice with ice-cold PBS, and resuspended in binding buffer at a density of 1 × 10^6^ cells/mL. The cellular suspensions were labeled with PE-conjugated Annexin V (5 μL) and 7-AAD (5 μL), followed by 15 minutes of incubation in the dark. Apoptotic profiles were quantified using flow cytometry (BD Biosciences, San Jose, CA) with subsequent data interpretation via FlowJo software (v10.8.1, FlowJo LLC).

Concurrently, treated cell populations in 6-well plates were fluorescently labeled with Hoechst 33342 (5 μg/mL) under light-protected conditions for 5 minutes at ambient temperature. Nuclear morphological alterations were visualized using a confocal laser-scanning microscope (Leica TCS SP8, 350 nm excitation/460 nm emission filters) to assess chromatin condensation and the formation of apoptotic bodies.

### 2.4 Western blotting

Cellular lysates were prepared using an ice-chilled RIPA lysis solution supplemented with 1 mM PMSF and a phosphatase inhibitor cocktail (Sangon Biotech, Shanghai, China). A BCA assay kit (Beyotime Biotechnology, China) was used to measure protein concentrations. Following SDS–PAGE separation, the protein were electrotransferred onto activated PVDF membranes. The membranes were then blocked with 5% nonfat dry milk in TBST for 60 minutes at ambient temperature. The membranes were probed overnight at 4 °C with primary antibodies diluted in blocking buffer, followed by incubation with horseradish peroxidase-linked secondary antibodies. Chemiluminescent signals were detected with a commercial ECL substrate (Thermo Fisher Scientific, MA, USA) and quantified with ImageLab software (Bio-Rad Laboratories). Antibodies were obtained from the following sources (1): Cell Signaling Technology (CST, MA, USA): cleaved caspase-3 (mAb, #9664; 1:1,000); AKT (mAb, #4691; 1:1,000); FOXO1 (mAb, #2880; 1:1,000); p-FOXO1 (mAb, #9464; 1:1,000); BIK (pAb, #4592; 1:1,000); TNFRSF9 (pAb, #62634; 1:1,000); phospho-AKT (Ser473) (mAb, #4060; 1:1,000) (2); Abcam (Cambridge, UK): GnRHR (pAb, ab183079; 1:1,000); cleaved PARP (mAb, ab32064; 1:1,000); and CIDEA (pAb, ab151577; 1:1,000).

### 2.5 siRNA and cell transfection

siRNAs were designed and synthesized by Shanghai GenePharma Co., Ltd. (Shanghai, China). Transfection procedures were performed using Lipofectamine 2000 (Thermo Fisher Scientific, MA, USA) according to standardized protocols. In brief, SKOV3 cells were seeded in six-well tissue culture plates at an initial concentration of 2×10^-5^ cells/well and cultured for 24 hours until they reached 40–50% confluence. Prior to transfection, the medium was replaced with serum/antibiotic-depleted RPMI-1640. For the transfection complexes, 75 pmol of siRNA and 5 μL of Lipofectamine 2000 were separately diluted in 125 μL of Opti-MEM-reduced serum medium and equilibrated at room temperature for 5 minutes. The diluted siRNA and Lipofectamine solutions were then mixed and incubated for 20 minutes to allow nanoparticle formation. Subsequently, 250 μL of the siRNA–lipid complex was added to each well; a 2 mL total volume was maintained. After 5 hours of incubation (5% CO_2_ humidified), the medium was replaced with fresh RPMI-1640 containing 10% FBS and 1% penicillin/strep.

### 2.6 Human apoptosis gene PCR array and qRT–PCR

SKOV3-ip cells were serum starved for 18 hours before cetrorelix/HCl treatment. After 48 hours of treatment, the RNA was isolated (RNeasy Kit, Qiagen) and reverse-transcribed (RT2 Kit, Qiagen) in strict adherence to standardized protocols. The expression profiles of the apoptosis-associated genes were analyzed with RT2 Profiler™ PCR Arrays (Qiagen), which enabled the systematic profiling of 84 apoptosis-related genes spanning multiple signaling pathways. Differentially expressed genes were validated using qRT–PCR (ABI 7000, Takara Bio). All primer sequences (Sangon Biotech) utilized for qRT–PCR are listed in **Supplementary Table S1**.

### 2.7 *In vivo* study

Female BALB/c nude mice (5 weeks old, 18–20 g, specific pathogen-free [SPF]) were acquired from Shanghai SLAC Laboratory Animal Co., Ltd. All animal procedures were executed in full compliance with China’s National Animal Welfare Guidelines and approved by the Animal Ethics Committee of Fudan University (Approval No. 2024–FCKYY–157).

For the *in vivo* study, cetrorelix was solubilized in 1 mol/L HCl, after which the pH was adjusted to 7.0 with an equal volume of 1 mol/L NaOH. The solution was then thinned with normal saline until the final concentration reached 100 μg/0.2 mL. For the HCl group, 1 mol/L HCl was adjusted to pH 7.0 with an equal amount of 1 mol/L NaOH and diluted in the same proportion as the cetrorelix group.

To establish xenograft models, 14 mice received subcutaneous injections of SKOV3 cell suspensions (1×10^7^ cells/mL) in the right scapular region. Six of these mice were implanted with CM-DiI fluorescently labeled cells (Thermo Fisher Scientific) for *in vivo* tracking. After seven days, the experimental group of mice (6 labeled + 8 unlabeled mice) was randomly assigned to one of two treatment groups: the cetrorelix group (n = 7) was subcutaneously injected daily with 100 μg/0.2 mL cetrorelix (13), and the HCl group received a subcutaneous injection of 200 μL of HCl daily. Using an *in vivo* imaging device, subcutaneous xenograft tumors in nude mice were tracked. Following 19 days of therapeutic intervention, the mice were humanely euthanized, and the xenograft tumors were immediately fixed in 4% PFA for histopathological analysis and TUNEL staining analyses. A comparative analysis of the TUNEL assay results and protein expression profiles (FOXO1, AKT/p-AKT, and GnRHR) between the two groups was performed using immunohistochemistry (IHC).

### 2.8 IHC and TUNEL staining

After being fixed in 4% PFA, the xenograft tumors were dehydrated through a graded ethanol series (70%-100%) and then paraffin-embedded. Following the generation of 5-μm-thick sections, immunostaining was conducted using a Histostain-Plus IHC Kit (NeoBioscience) to detect the following target antigens: GnRHR (1:300), p-AKT (1:50), AKT (1:200) and FOXO1 (1:50). TUNEL staining was subsequently conducted with a TUNEL Apoptosis Assay Kit (Roche Diagnostics) following standardized protocols. Semiquantitative analysis was performed independently by two pathologists who were blinded to all patient information. The immunoreactive score (IRS) system, as previously described (14, 15), was applied consistently across all markers evaluated in this study. The positivity cut-off was established based on the median of IRS values (AKT ≥4, GnRHR ≥6, FOXO1 ≥ 2). The IRS was determined by calculating the product of the semiquantitative intensity (SI) score and positive proportion (PP). The SI score was determined as follows: 0 (no staining), 1 (faint staining), 2 (intermediate staining), and 3 (intense staining). The PP score was determined as follows: 0 (0%), 1 (1–10%), 2 (11–50%), 3 (51–75%), and 4 (>75%). To ensure scoring reproducibility, the inter-observer agreement was assessed on a random subset of 20% of the slides using Cohen’s kappa statistic, which showed excellent agreement (κ = 0.85). Any initial scoring discrepancies were resolved through simultaneous re-examination under a multi-headed microscope until a consensus was achieved.

### 2.9 Clinical data

This study was approved by the Ethics Committee of the Obstetrics and Gynecology Hospital of Fudan University (No. 2024-155). All study participants provided written informed consent. We conducted a retrospective analysis of EOC patients who underwent primary cytoreductive surgery with staging procedures at our institution and whose tumor biospecimens were accessible between January 2013 and December 2015. The exclusion criteria included (1) any preoperative systemic treatment history (2); nonepithelial ovarian malignancies; and (3) borderline ovarian tumors.

Clinicopathological data were obtained from medical records. Patient characteristics, including age, FIGO stage (2014 criteria), operation date, and longitudinal disease status, were recorded. Continuous postoperative surveillance was maintained through August 31, 2025. Overall survival (OS) was defined as the duration from primary surgical treatment to either mortality events or study termination, with surviving patients censored at the last follow-up date. A total of 104 serous adenocarcinoma (SAC) patients were identified, and their clinical features are shown in **Supplementary Table S2**. Immunohistochemical evaluation was performed on tissue microarrays using standard immunohistochemistry methods.

### 2.10 Statistical analyses

Statistical analyses were conducted using Stata 14.0 and GraphPad Prism 6.0. Continuous variables are presented as the means ± standard deviations; between-group comparisons were conducted via Student’s t test (two groups) or ANOVA (multiple groups). The categorical variables were analyzed using the χ² test or Fisher’s exact test where applicable. OS was evaluated with the Kaplan–Meier method, log-rank tests, and Cox regression analyses for univariate and multivariate analyses. *p <*0.05 was considered to indicate statistical significance.

## 3 Results

### 3.1 Cetrorelix induces EOC cell apoptosis *in vitro*

**Figure 1:**
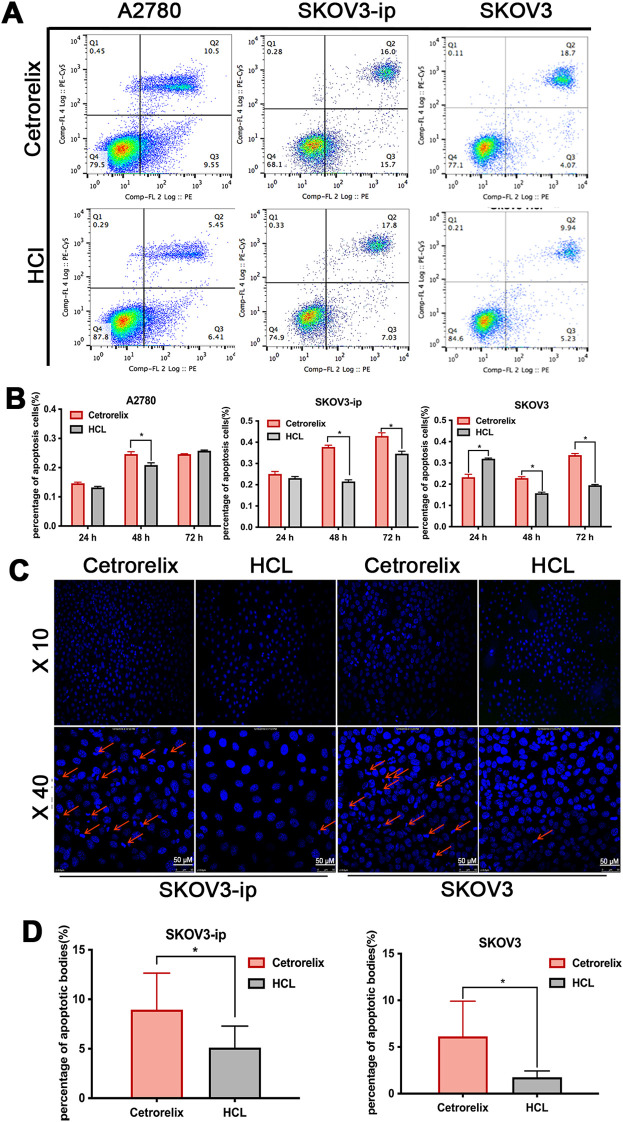
Cetrorelix induced EOC apoptosis *in vitro.* Representative dot plots (for 48 h) **(A)** and quantification **(B)** of apoptotic SKOV3, SKOV3-ip, and A2780 cells after treatment with 100μM cetrorelix at different time points. Representative micrographs **(C)** of Hoechst staining analysis and quantification **(D)** of apoptotic bodies after treatment with 100μM cetrorelix for 48 hours in SKOV3-ip and SKOV3 cells. ×10 (upper panel) and ×40 (lower panel) original magnifications. The red arrows indicate apoptotic body formation (*p <0.05).

**Figure 2:**
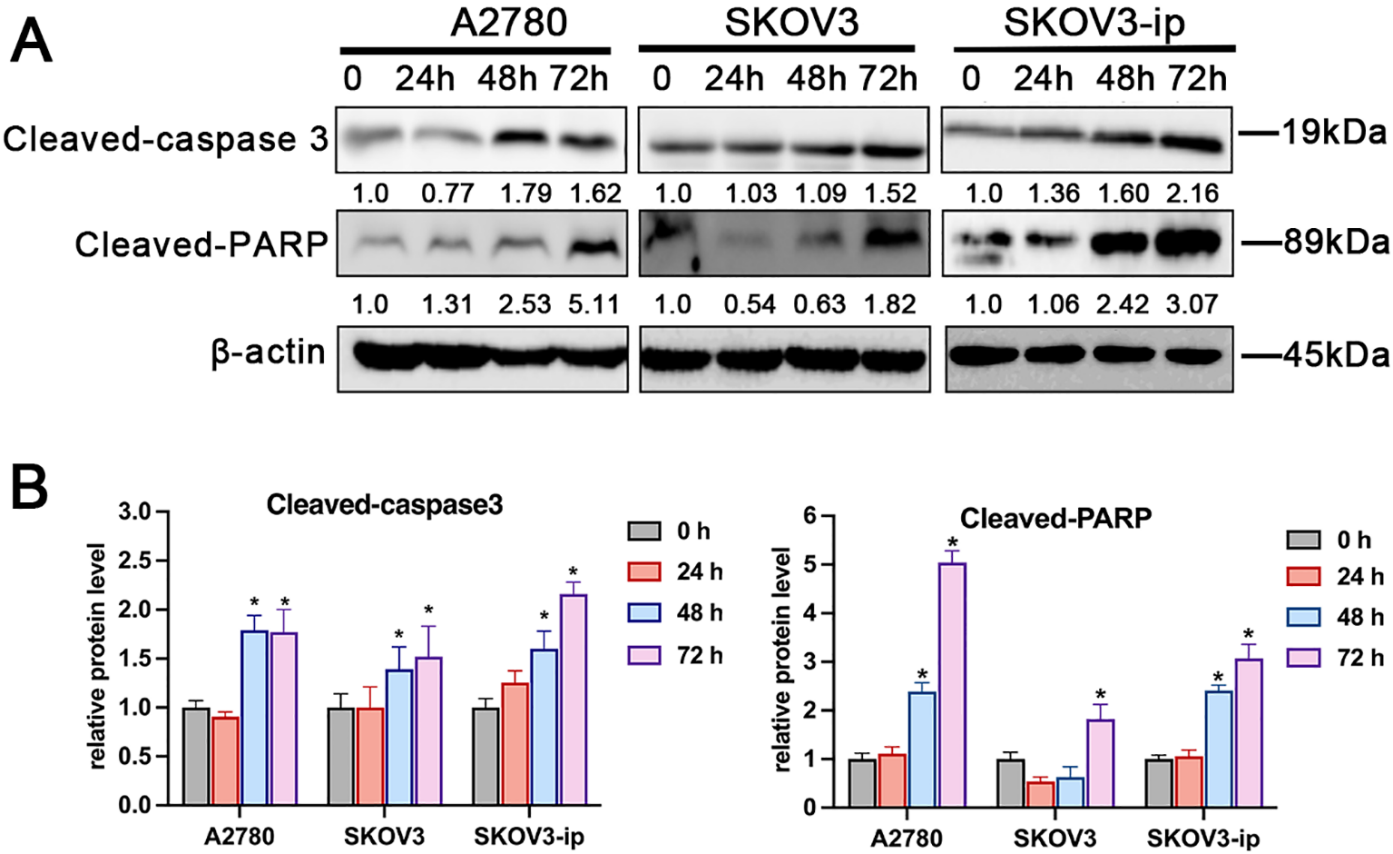
Western blot **(A)** and quantification **(B)** of cleaved caspase-3 and cleaved PARP expression after treatment with 100μM cetrorelix in SKOV3, SKOV3-ip, and A2780 cells at different timepoints (*p <0.05).

All A2780, SKOV3 and SKOV3-ip cells exhibited GnRHR expression (**Supplementary Figure S1A**). According to flow cytometry, compared with that in the control group, the percentage of apoptotic SKOV3-ip cells increased after treatment with varying cetrorelix concentrations for 48 and 72 hours (*p* < 0.05; **Supplementary Figures S1B, C**). As shown in **Figures 1A, B**, SKOV3-ip, SKOV3, and A2780 cells presented significantly higher total apoptosis rates after treatment with 100μM cetrorelix. Similarly, Hoechst staining revealed greater apoptotic body formation in cetrorelix-exposed SKOV3-ip and SKOV3 cells than in vehicle-treated cells *(p* < 0.05; **Figures 1C, D**). Complementary western blot analyses further confirmed that cetrorelix significantly promoted caspase 3 and PARP cleavage/activation *(p* < 0.05; **Figures 2A, B**), which are hallmarks of apoptosis. Overall, these findings demonstrate that cetrorelix induces EOC apoptosis.

### 3.2 Cetrorelix modulates EOC apoptosis by increasing FOXO1 expression via the PI3K/AKT signaling pathway

**Figure 3:**
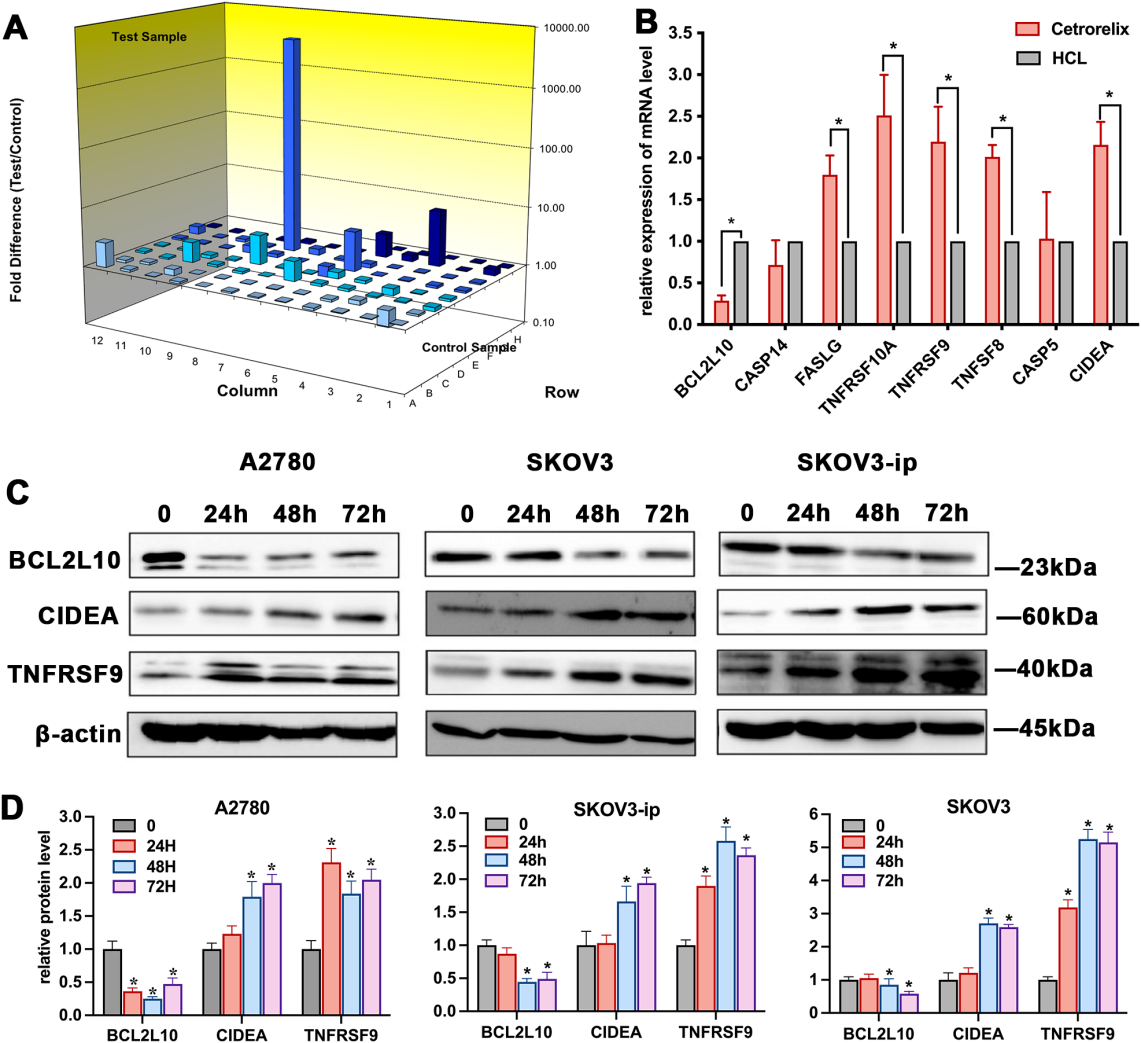
Expression of human apoptosis-related genes regulated by cetrorelix in EOC cells. Human apoptosis gene PCR array **(A)**, qRT–PCR **(B)**, western blot analysis **(C)** and quantification **(D)** of downstream molecular events involving cetrorelix and EOC apoptosis (*p <0.05).

According to the human apoptosis gene PCR array (**Figure 3A**), we detected 8 downstream molecular processes (fold change ≥1.5) influenced by cetrorelix in the SKOV3-ip-Cetrorelix group (**Supplementary Table S3**). qRT–PCR and western blot analyses validated the expression of these genes, including members of the tumor necrosis factor (TNF) and TNF receptor superfamilies (such as TNFRSF9, TNFSF8, and TNFRSF10A) (**Figures 3B–D**). These results were consistent with those of the PCR array analysis.

**Figure 4:**
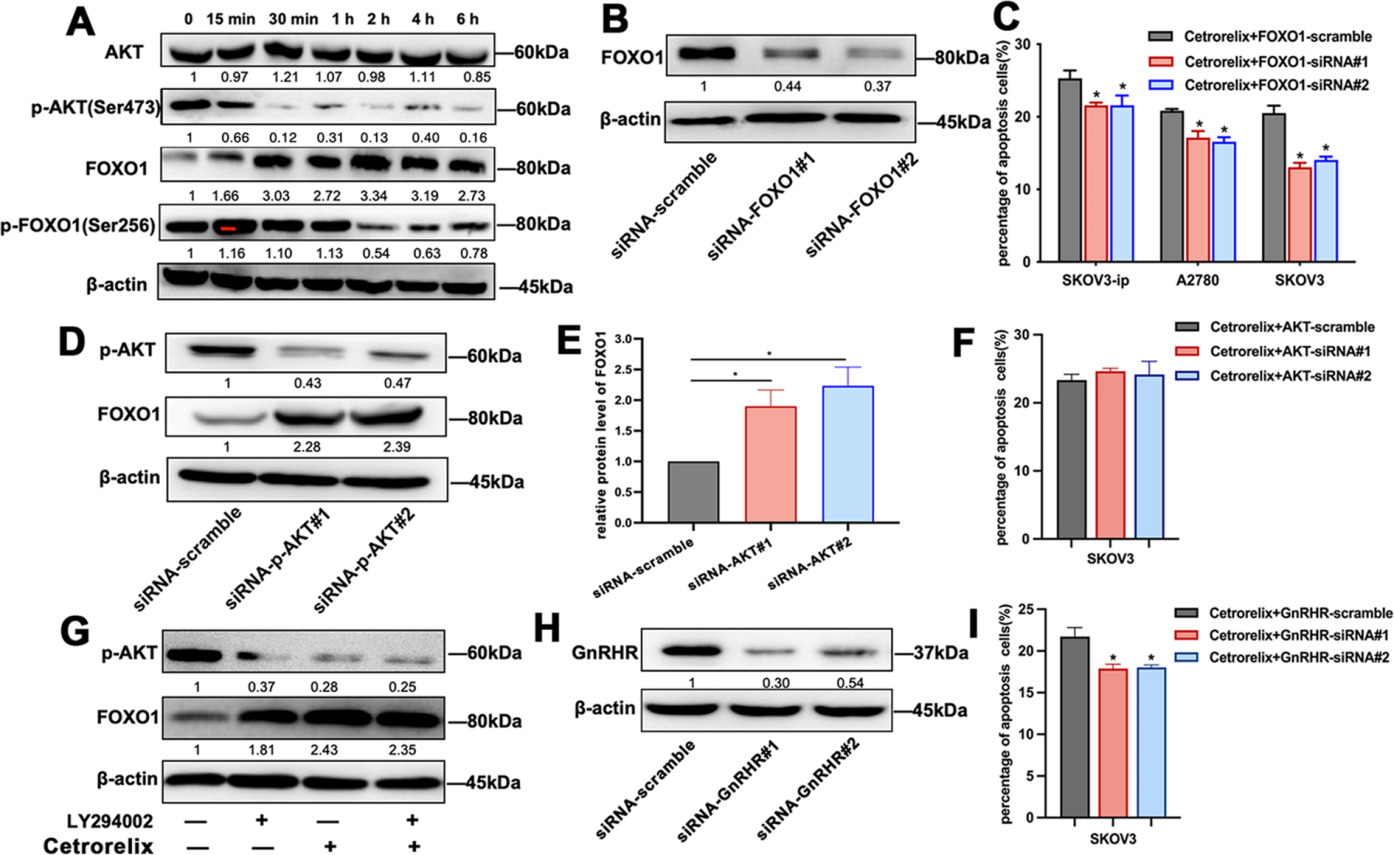
Cetrorelix upregulates FOXO1 expression through the PI3K/AKT signaling pathway. **(A)** Western blot analysis of FOXO1, AKT and p-AKT expression in SKOV3 cells after treatment with 100μM cetrorelix at different time points. After FOXO1 was knocked down using siRNA **(B)**, flow cytometric analysis revealed that the promotion of apoptosis by cetrorelix was abrogated **(C)**. Western blot analysis **(D)** and quantification **(E)** revealed that FOXO1 expression was increased by AKT-siRNA in SKOV3 cells. **(F)** Flow cytometry reveals no additive apoptosis when cetrorelix is combined with AKT-siRNA, supporting that they act through the same pathway. **(G)** Western blot analysis shows that both cetrorelix and the PI3K inhibitor LY294002 suppress p-AKT and upregulate FOXO1. GnRHR knockdown **(H)** abrogates the pro-apoptotic response to cetrorelix, as quantified by flow cytometry **(I)** (*p <0.05).

According to previous reports, FOXO1 regulates the expression of TNF and its receptors (15). To explore the potential role of FOXO1 in cetrorelix-induced apoptosis of EOC cells, we detected FOXO1 expression following treatment with 100μM cetrorelix. As demonstrated in **Figure 4A**, compared with vehicle treatment, cetrorelix treatment significantly upregulated FOXO1 expression and downregulated p-FOXO1 level in EOC cells. The qRT-PCR results showed significantly elevated levels of the FOXO1 downstream targets BIM, PUMA, and FasL (**Figure 3B** and **Supplementary Figure S2**), further supporting that cetrorelix enhances FOXO1 transcriptional activity. Importantly, FOXO1 silencing via siRNA transfection significantly suppressed the cetrorelix-mediated induction of apoptosis (*p* < 0.05; **Figures 4B, C**), indicating that FOXO1 plays an essential role in this process.

Given that FOXO1 functions as a substrate in the PI3K/AKT signaling axis, we further investigated the modulation of AKT signaling. Cetrorelix specifically downregulated p-AKT (Ser473) without affecting total AKT expression (**Figure 4A**). Complementary experiments using AKT-siRNA revealed that AKT knockdown significantly increased FOXO1 expression (*p* < 0.05; **Figures 4D, E**). Furthermore, when cetrorelix was combined with the PI3K inhibitor LY294002, we observed that LY294002 mimicked the suppressive effect of cetrorelix on AKT phosphorylation and the subsequent upregulation of FOXO1(**Figure 4G**). Notably, AKT-siRNA did not lead to any further enhancement of apoptosis, supporting the conclusion that cetrorelix exerts its effects primarily through the PI3K/AKT pathway (**Figure 4F**). To experimentally verify whether the pro-apoptotic effect of cetrorelix is specifically mediated through GnRHR, we employed siRNA to knockdown GnRHR expression in SKOV3 cell (**Figure 4H**). The flow cytometry showed that knockdown of GnRHR abrogated cetrorelix-induced apoptosis, demonstrating that the pro-apoptotic effect of cetrorelix is specifically mediated through GnRHR (**Figure 4I**). Collectively, these findings suggest that cetrorelix induces EOC cell apoptosis via PI3K/AKT-dependent FOXO1 activation.

### 3.3 *In vivo* evaluation of cetrorelix in a nude mouse subcutaneous xenograft model

**Figure 5:**
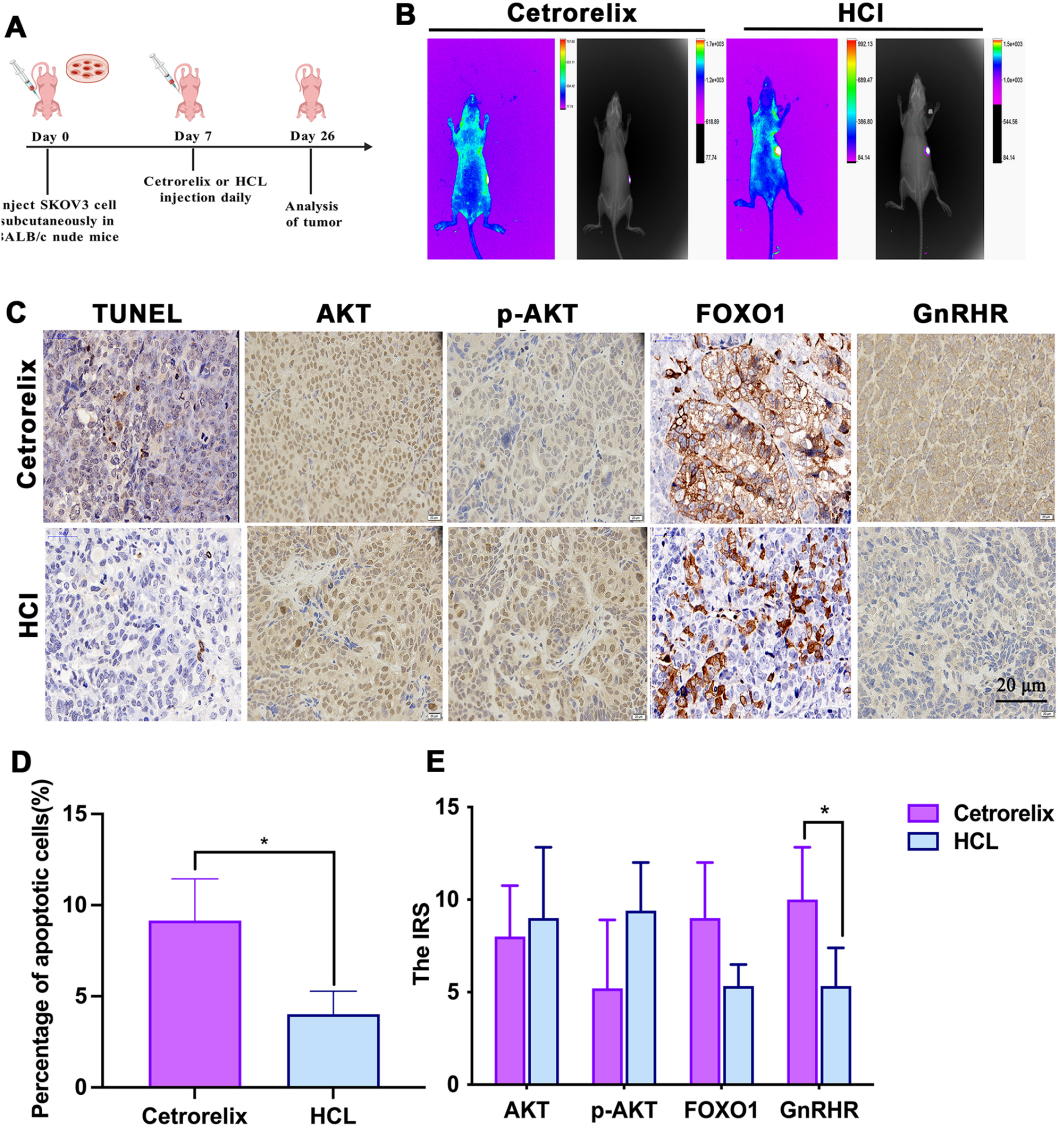
*In vivo* evaluation of the cetrorelix in a nude mouse subcutaneous xenograft model. **(A)** Schematic illustration of the experimental design used to assess the proapoptotic effect of cetrorelix. SKOV3 cells (1×10^7^ cells/mL) were subcutaneously injected into the right scapular region of female BALB/c nude mice. After seven days, the mice were randomized into two treatment cohorts accordingly (n = 7 for each group) and given drug treatment as follows: the HCl group (200 μL of HCl daily, subcutaneous injection) or the cetrorelix group (100 μg/0.2 mL cetrorelix daily, subcutaneous injection). Treatment was stopped on day 26, and the tumors were analyzed. **(B)***In vivo* images of subcutaneous xenograft tumors in nude mice after 7 days of treatment with cetrorelix. **(C)** TUNEL staining and immunohistochemistry analysis of AKT, p-AKT, FOXO1, and GnRHR protein expression in the Cetrorelix and HCl groups. Original magnification, ×40. Quantification of apoptotic cells **(D)** and the IRSs **(E)** of AKT, p-AKT, FOXO1, and GnRHR in the two groups (*p <0.05).

SKOV3 cells were injected into BALB/c nude mice, and cetrorelix or HCl was administered to the mice (**Figure 5A**). The successful establishment of the subcutaneous xenograft tumor model is shown in **Figure 5B**. TUNEL analysis revealed that cetrorelix markedly increased the proportion of apoptotic cells in the xenograft tissues (**Figures 5C, D**). The IHC results demonstrated that, compared with that in the HCl group, GnRHR expression was markedly increased in the cetrorelix group (p <0.05). p-AKT expression tended to decrease, whereas FOXO1 expression increased in the cetrorelix group. However, the differences were not significant. Moreover, there was no notable difference in AKT expression between the two groups (*p* > 0.05; **Figures 5C, E**). Overall, these *in vivo* observations of PI3K/AKT/FOXO1 signaling components aligned closely with the corresponding *in vitro* findings.

### 3.4 Correlations of GnRHR/AKT/FOXO1 expression with clinicopathological features in SAC patients

**Figure 6:**
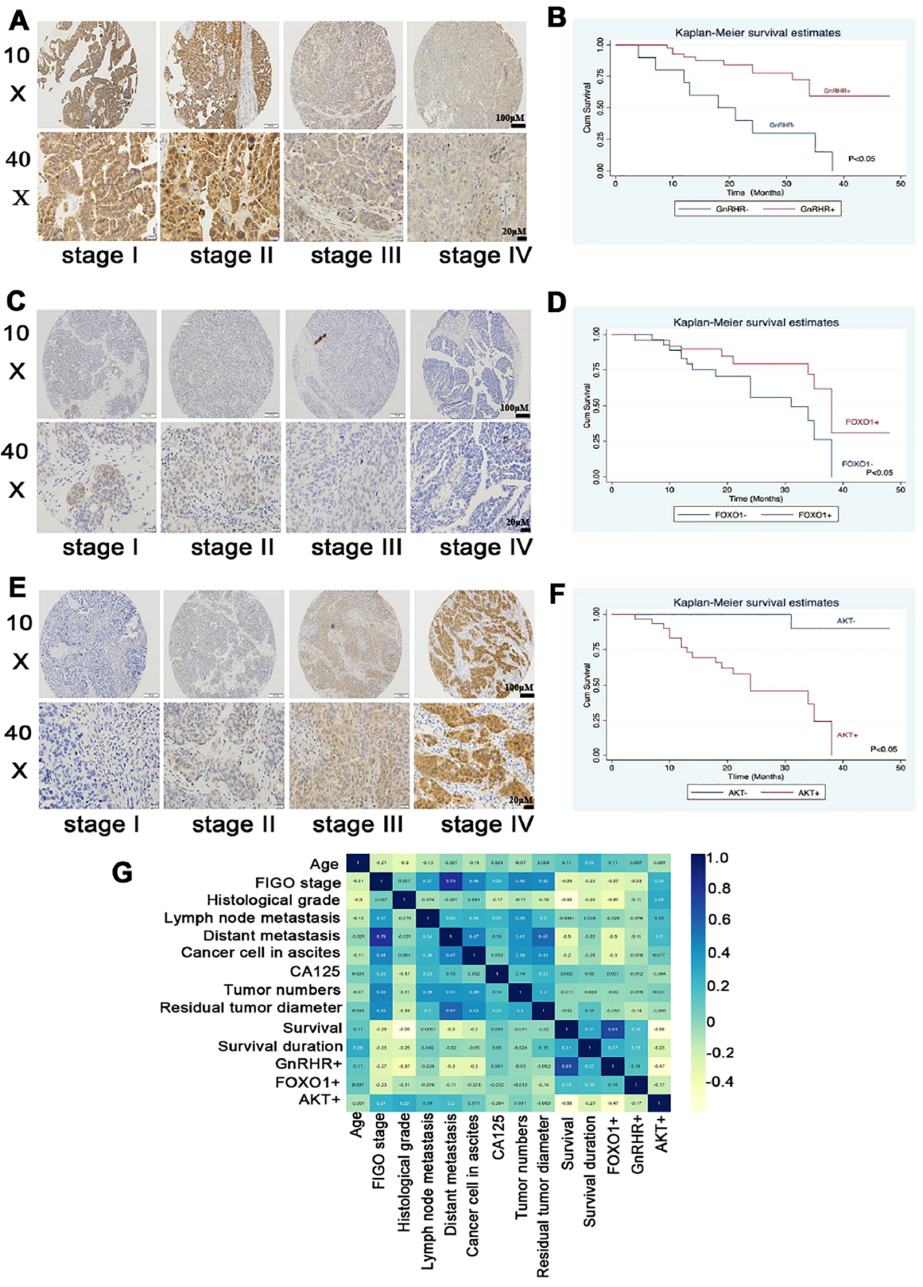
GnRHR/FOXO1/AKT expression levels in SAC tissues and their correlations with patient clinicopathological features. Representative immunohistochemical images demonstrating differential GnRHR **(A)**, FOXO1 **(C)** and AKT **(E)** expression patterns in SAC samples stratified by FIGO stage. ×10 (upper panel) and x40 (lower panel) original magnification. Kaplan–Meier overall survival curves illustrating the prognostic significance of GnRHR **(B)**, FOXO1 **(D)** and AKT **(F)** expression. **(G)** Pearson correlation analysis of feature relationships. The *p* value was calculated using a log-rank test.

The relationships between FOXO1/AKT/GnRHR expression patterns and clinicopathological characteristics were analyzed using immunohistochemical data from tissue microarrays of 104 SAC patients. As shown in **Figure 6A**, GnRHR expression was significantly lower in advanced-stage disease (FIGO stages III–IV) compared with early-stage disease (FIGO stages I–II; *p <*0.05). Notably, patients who presented with prognostic risk factors, including malignant ascites, lymph node involvement, and distant metastases, presented substantially reduced GnRHR expression levels (*p* < 0.05; **Figure 6G**, **Table 1**). This clinical correlation was further supported by survival analysis, which revealed a superior prognosis in GnRHR-positive patients (**Figure 6B**). These findings collectively suggest that preserved GnRHR expression may serve as a favorable prognostic biomarker in EOC and highlight the potential therapeutic implications of GnRH antagonist-mediated pathway modulation.

**Table 1:** Correlations between the clinicopathologic characteristics of patients with SAC and GnRHR, FOXO1 and AKT expression.

Variables	N	GnRHR				FOXO1				AKT			
		positive	negative	IRS	*P* value	positive	negative	IRS	*P* value	positive	negative	IRS	*P* value
Age (years)													
<50	30	13	17	6.23 ± 3.41	0.46	11	19	1.3 ± 1.68	0.16	16	14	4.5 ± 3.30	0.282
≥50	74	40	34	6.82 ± 3.78		38	36	2.01 ± 2.39		45	29	5.35 ± 3.76	
Histological grade													
High grade	79	43	36	6.86 ± 3.54	0.31	40	39	2 ± 2.40	0.14	40	39	4.55 ± 3.75	0.006*
Low grade	25	15	10	6.0 ± 4.08		9	16	1.24 ± 1.50		21	4	6.84 ± 2.65	
FIGO stage													
I-II	41	31	10	8.51 ± 3.49	<0.001*	23	18	2.48 ± 2.81	0.013*	18	23	3.44 ± 3.09	0.001*
III-IV	63	22	41	5.44 ± 3.29		26	37	1.38 ± 1.63		43	20	6.19 ± 3.58	
Lymph node metastasis													
Absent	77	45	32	7.22 ± 3.79	0.007*	37	40	1.96 ± 2.38	0.27	41	36	4.77 ± 3.81	0.11
Present	27	8	19	5.03 ± 2.81		12	15	1.40 ± 1.70		20	7	6.07 ± 2.94	
Distant metastasis													
Absent	48	33	15	8.02 ± 3.57	0.0003*	25	23	2.23 ± 2.70	0.08	23	25	3.68 ± 3.07	0.002*
Present	56	36	20	5.48 ± 3.36		24	32	1.46 ± 1.68		38	18	6.32 ± 3.67	
Cancer cell in ascites													
Absent	68	42	26	7.48 ± 3.53	0.0012*	32	36	1.96 ± 2.46	0.39	38	30	4.53 ± 3.52	0.026*
Present	36	11	25	5.08 ± 3.46		17	19	1.56 ± 1.70		23	13	6.19 ± 3.65	
Survival duration													
≤2 years	30	9	21	4.6 ± 3.20	0.0002*	9	21	1 ± 1.46	0.017*	30	0	8.07 ± 1.98	0.001*
>2 years	74	44	30	7.48 ± 3.53		40	32	2.14 ± 2.40		31	43	3.91 ± 3.47	

IRS, immunoreactive score; *p < 0.05, t test.

A parallel expression pattern emerged for FOXO1, which was expressed at significantly higher levels in FIGO stage I-II tumors compared with FIGO stage III-IV tumors (*p <*0.05; **Figure 6C**; **Table 1**). Consistent with the GnRHR findings, positive FOXO1 expression correlated with favorable clinical outcomes (**Figures 6D, G**). Conversely, AKT expression was significantly greater in patients with FIGO stage III-IV disease than in those with stage I-II disease (*p <*0.05; **Figure 6E**; **Table 1**). In patients with distant metastasis or malignant ascites, AKT expression was markedly elevated (*p <*0.05; **Table 1**). Patients with positive AKT expression had a poorer prognosis than those with negative AKT expression did (*p <*0.05; **Figures 6F, G**).

### 3.5 GnRHR positivity and AKT negativity were identified as independent risk factors for improved overall survival

Using univariate Cox regression, key variables affecting the overall survival (OS) of SAC patients were screened. These included the expression of GnRHR (HR: 0.147 (0.075-0.288); *p <*0.05), FOXO1 (HR: 0.319 (0.122-0.834); *p <*0.05), and AKT (HR: 15.217 (3.659-63.295); *p <*0.05); FIGO stage III/IV (HR: 2.851 (1.295-6.274); *p <*0.05); high-grade pathological type (HR: 5.13 (2.692-9.780); and distant metastasis (HR: 2.373 (1.143-4.927); *p <*0.05). On the basis of the multivariable analysis, higher GnRHR expression (HR = 0.309, 95% CI 0.146-0.655; *p* < 0.05) was an independent protective factor, whereas elevated AKT expression (HR = 5.325; 95% CI 1.159-24.468; *p* < 0.05), high-grade pathological type (HR = 9.597; 95% CI 3.885-23.709; *p* < 0.05) and advanced FIGO stage (HR = 5.773; 95% CI 1.029-32.389; *p* < 0.05) were independent risk factors for overall survival (**Table 2**).

**Table 2:** Univariate and multivariable survival analysis of factors associated with OS in SAC patients.

Prognostic variables	Univariable analysis		Multivariable analysis	
	HR (95% CI)	*p*	HR (95% CI)	*p*
GnRHR				
GnRHR+	0.147 (0.075-0.288)	<0.001*	0.309 (0.146-0.655)	0.002*
GnRHR-	—	—		
FOXO1				
FOXO1+	0.319 (0.122-0.834)	0.02*	0.895 (0.327-2.450)	0.829
FOXO1-	—	—		
AKT				
AKT+	15.217 (3.659-63.295)	<0.001*	5.325 (1.159-24.468)	0.032*
AKT-	—	—		
Age(years)				
<50	0.778 (0.373-1.623)	0.504		
≥50	—	—		
Histological grade				
High grade	5.13 (2.692-9.780)	0.001*	9.597 (3.885-23.709)	0.001*
Low grade	—	—		
FIGO stage				
III-IV	2.851 (1.295-6.274)	0.009*	5.773 (1.029-32.389)	0.046*
I-II	—	—		
Lymph node metastasis				
Present	1.017 (0.490-2.107)	0.964		
Absent	—	—		
Distant metastasis				
Present	2.373 (1.143-4.927)	0.020*	1.244 (0.256-6.042)	0.787
Absent	—	—		
Cancer cell in ascites				
Present	1.778 (0.938-3.364)	0.077		
Absent	—	—		
Residual tumor diameter (cm)				
≥1	1.393(0.635-3.059)	0.408		
<1	—	—		

HR, Hazard ratio; CI, confidence interval; *p <0.05.

## 4 Discussion

Several preliminary reports have explored the function and biological mechanism of GnRH antagonists in cancer apoptosis. Claudio Festuccia et al. reported that ozarelix, a GnRH antagonist, induced apoptosis through caspase-8-dependent activation of caspase-3 accompanied by the downregulation of c-FLIP (L) in prostate cancer cells (16). Carsten Gründker et al. reported that a GnRH type II antagonist induced apoptosis in breast cancer models via stress-activated MAPK p38 activation, with a reduction in the mitochondrial membrane potential both *in vivo* and *in vitro* (17). We discovered that cetrorelix induced EOC apoptosis in both cellular and animal models, which was corroborated by various methodologies across distinct EOC cell lines. Furthermore, we demonstrated that GnRHR expression was markedly elevated in patients with FIGO stage I–II disease compared with patients with FIGO stage III–IV disease and that patients with positive GnRHR expression had a better prognosis than those with negative GnRHR expression did. These results confirmed the anticancer value of GnRH antagonist/GnRHR in EOC treatment, particularly given its relatively low drug toxicity and potential adverse effects compared with those of chemotherapy.

To explore the molecular processes responsible for cetrorelix-induced apoptosis in EOC cells, we used a human apoptosis gene PCR array. Our analysis revealed that cetrorelix induced EOC apoptosis by regulating the expression of TNF and its receptor superfamily members (such as TNFRSF9, TNFSF8, and TNFRSF10A), which are regulated by FOXO1 (18, 19). FOXO1, a member of the forkhead protein O family, functions as a tumor suppressor by modulating cell differentiation and proliferation and by participating in DNA damage repair. Indeed, its multifaceted regulatory roles have been well documented in prostate cancer (20), pancreatic disease (21) and breast cancer (22, 23). With respect to ovarian cancer, Roya Ghaffarnia et al. reported that 10058-F4 exerts anticancer effects via the upregulation of the expression of FOXO transcription factors and their downstream genes, such as FasL, thereby inducing apoptosis in ovarian cancer cells (24). Our clinical correlation analysis revealed that patients with positive FOXO1 expression had a better prognosis, suggesting its potential tumor-suppressive function in EOC pathogenesis. Consistent with our hypothesis, cetrorelix significantly upregulated FOXO1 expression and downregulated p-FOXO1 levels. The significant upregulation of FOXO1 downstream targets (BIM, PUMA, and FasL) at the mRNA level is consistent with enhanced FOXO1 transcriptional activity upon cetrorelix treatment. Moreover, the FOXO1-siRNA abolished the proapoptotic effect of the cetrorelix on EOC cells. These findings collectively demonstrate that cetrorelix induces EOC apoptosis through FOXO1-mediated regulatory mechanisms. While our *in vitro* data suggest that cetrorelix activates FOXO1, the *in vivo* evidence, based on IHC staining, is indicative of a trend rather than a statistically significant change. Future studies quantifying FOXO1 protein levels or its nuclear localization are needed to definitively establish its role in the anti-tumor effects observed *in vivo*.

The PI3K/AKT pathway functions as a master regulator of processes such as proliferation, survival signaling, migratory capacity, metabolic reprogramming, and cytoskeletal reorganization (25). Previous evidence has indicated that the phosphorylation of FOXO1, a key determinant of its nucleocytoplasmic shuttling and subsequent biological functions, is predominantly regulated by the PI3K/AKT pathway (26). AKT-mediated FOXO1 phosphorylation results in the retention of FOXO1 in the cytoplasm and subsequently suppresses its transcriptional activity. Notably, the PI3K/AKT/FOXO1 pathway has been implicated in the regulation of apoptosis (27, 28). On the basis of this evidence, we propose that cetrorelix induces EOC apoptosis through transcriptional activation of FOXO1, which is mediated by suppression of the PI3K/AKT signaling axis. As anticipated, cetrorelix suppressed PI3K/AKT signaling activation, and FOXO1 upregulation was dependent on this pathway. Moreover, a decrease in phosphorylated FOXO1 levels was detected, which subsequently triggered the upregulation of the expression of FOXO1-dependent downstream transcriptional targets, such as TNF and its receptor. Furthermore, the finding that the PI3K inhibitor LY294002 mimicked the effect of cetrorelix and that the combination treatment yielded no additive pro-apoptotic effect critically positions the PI3K/AKT axis as the primary downstream signaling module.

In our study, we used total−AKT and p−AKT (Ser473) antibodies to assess the overall suppression of the PI3K/AKT signaling pathway by cetrorelix. While p−AKT (Ser473) can, to some extent, reflect the general pathway activity, the three AKT isoforms (AKT1, AKT2 and AKT3) are not functionally equivalent in ovarian cancer. Therefore, the lack of isoform-specific data should be clearly framed as a limitation of the present study and a direction for future work. Further delineation of their individual contributions would provide deeper insights into the mechanism of action of cetrorelix. Existing literature has reported that AKT1 is the predominant isoform driving proliferation in EOC cells and is frequently activated in ovarian cancer (29). AKT2 is linked to metabolic re−programming and invasion (30), and AKT3 correlates with cancer growth, aggressiveness and chemoresistance in ovarian cancer (31), suggesting that each isoform may contribute uniquely to the cetrorelix response. To clarify their individual roles, future work will (i) perform isoform−specific Western blots for total and p−Ser473 AKT1/2/3, (ii) use siRNA to knock down each isoform and assess apoptosis after cetrorelix treatment, and (iii) test isoform−selective AKT inhibitors in combination with cetrorelix for synergistic effects. These experiments will allow us to pinpoint which AKT isoform(s) mediate the pro−apoptotic activity of cetrorelix.

**Figure 7:**
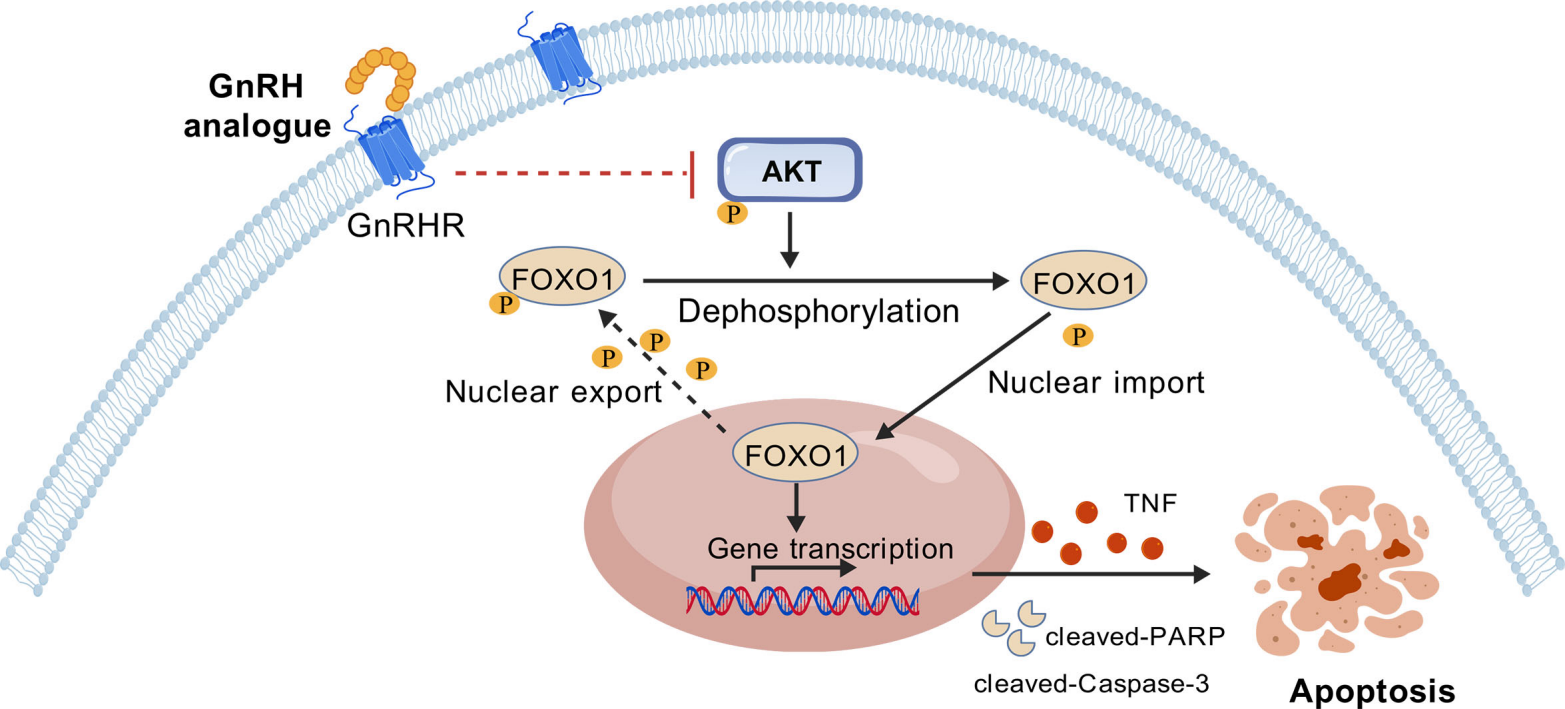
Schematic of the mechanisms by which cetrorelix triggers EOC apoptosis.

Moreover, the dependence on GnRHR was demonstrated by the abrogation of apoptosis upon receptor knockdown. Therefore, we conclude that cetrorelix induces apoptosis primarily via GnRHR-mediated inactivation of the PI3K/AKT pathway and the consequent activation of FOXO1(**Figure 7**). Importantly, *in vivo* experiments revealed expression patterns of key PI3K/AKT/FOXO1 pathway components that were concordant with the *in vitro* observations. Moreover, the precise molecular mechanism through which cetrorelix affects the PI3K/AKT pathway deserves further in-depth exploration. As a GnRH receptor antagonist, cetrorelix blocks the canonical signaling cascade, preventing the GnRH-induced increase in intracellular Ca^2+^ and cAMP and the subsequent activation of PI3K upstream kinases PKC and PKA (32). Furthermore, GnRH signaling in ovarian−cancer cells frequently cross−activates EGFR/HER2, providing an additional PI3K input; antagonism of GnRH−R therefore reduces this RTK−driven stimulu (33). These proposed mechanisms will guide our future investigations to definitively elucidate the inhibitory actions of cetrorelix. Intriguingly, our prior work identified goserelin, a GnRH agonist, as a pro-apoptotic agent in EOC that exerts effects via the PI3K/AKT/FOXO1 axis (34), mirroring the cetrorelix mechanism despite their opposing pharmacological classifications. This phenomenon may be explained by the principle of ‘ligand-biased signaling’ at the GPCR. We hypothesize that chronic agonist exposure may induce a biased signaling state that selectively dampens survival pathways, whereas antagonists achieve a similar outcome through comprehensive receptor blockade. Both mechanisms appear to converge on the critical inactivation of PI3K-AKT and subsequent activation of FOXO1, highlighting this axis as a central node for GnRH-mediated anti-tumor effects. Future work will focus on validating this model through detailed profiling of the distinct signaling fingerprints of these ligands. And whether other GnRH antagonists or agonists exert their effects through an identical mechanistic pathway in ovarian cancer requires further comparative studies.

Our study further investigated the clinical significance of key signaling components in EOC. Research has revealed elevated FOXO1 expression in DOX-resistant triple-negative breast cancer (TNBC) cells, where it maintains redox homeostasis in chemotherapy-resistant cells (22). Notably, Chen et al. demonstrated that elevated GnRHR expression is associated with increased disease-free survival in TNBC patients (35). Our data demonstrated that reduced FOXO1 and GnRHR expression were significantly correlated with advanced FIGO stage and distant metastasis, whereas reduced AKT expression was correlated with these features. Multivariate analysis revealed that high AKT expression and low GnRHR expression were independent risk factors for poor overall survival. Strikingly, GnRHR-negative tumors were strongly correlated with FIGO progression, lymph node metastasis, and distant dissemination, further reinforcing its role as a predictor of clinical outcomes. These findings suggest that GnRH antagonists may exhibit optimal efficacy in early-stage EOC. The pronounced association between GnRHR status and metastatic behavior warrants investigation into the anti-invasive and anti-migratory effects of GnRH antagonists on EOC cells. Taken together, these results suggest that GnRHR, FOXO1 and AKT are potentially dynamic regulators of tumor behavior and potentially represent promising therapeutic targets and prognostic indicators for EOC. Moreover, these experimental and clinical findings demonstrate remarkable consistency in molecular pathway regulation and prognostic relevance. Mechanistically, cetrorelix-induced FOXO1 activation through PI3K/AKT suppression aligns with clinical observations in which GnRHR or FOXO1 positivity and AKT negativity correlate with favorable outcomes. This concordance between cellular pathway modulation and patient stratification biomarkers supports the biological plausibility of the mechanism of action of cetrorelix in EOC management.

While this work provides evidence for the anti-tumor mechanism of cetrorelix, several limitations should be acknowledged. Firstly, the clinical relevance of the biomarker findings is constrained by the cohort size and requires validation in larger populations. Secondly, while cetrorelix demonstrated pro-apoptotic effects in our xenograft model, the *in vivo* observations were made over a relatively short treatment period. The long-term therapeutic efficacy, its impact on survival in mice and lasting impact on tumor regression remain unknown and warrant investigation in studies with extended follow-up. Thirdly, a major limitation of our study is that it does not address the potential influence of the hormonal microenvironment, particularly estrogen signaling, on cetrorelix’s efficacy. The efficacy of a GnRH antagonist in a hormone-sensitive cancer like EOC is inherently tied to endocrine context. Our mechanistic model, established under standard culture and xenograft conditions, may not fully represent the drug’s activity in the presence of fluctuating estrogen levels. Consequently, elucidating cetrorelix’s activity under defined hormonal conditions is not merely a future direction but a prerequisite for a comprehensive understanding of its mechanism of action. Finally, our study primarily focused on the PI3K/AKT–FOXO1 axis, and may overlook additional mechanisms that contribute to cetrorelix’s anti-tumor activity. A more comprehensive analysis of signaling networks will be valuable to fully understand cetrorelix’s effects.

In summary, our findings reveal a previously unrecognized mechanism by which cetrorelix induces apoptosis through the modulation of the PI3K/AKT–FOXO1 signaling pathway in EOC cells. GnRHR/FOXO1/AKT, key components in the signaling pathway, may be emerging therapeutic targets for EOC. Furthermore, these findings highlight the potential of GnRH antagonists as promising antitumor candidates, although translational and clinical studies are needed to assess their clinical value.

## Data Availability

The raw data supporting the conclusions of this article will be made available by the authors, without undue reservation.
