# Supplementary material for: Cetrorelix promotes cell apoptosis via the PI3K–AKT–FOXO1 pathway in epithelial ovarian cancer
Source: Front Oncol. 2025 Dec 12;15:1631576. doi: 10.3389/fonc.2025.1631576 (PMC12740856; doi:10.3389/fonc.2025.1631576)
Supplement: Supplementary file 1 [file DataSheet1.docx]

Supplementary Material

# Supplementary Figures and Tables

For more information on Supplementary Material and for details on the different file types accepted, please see [here](https://www.frontiersin.org/guidelines/author-guidelines#supplementary-material).

## Supplementary Figures


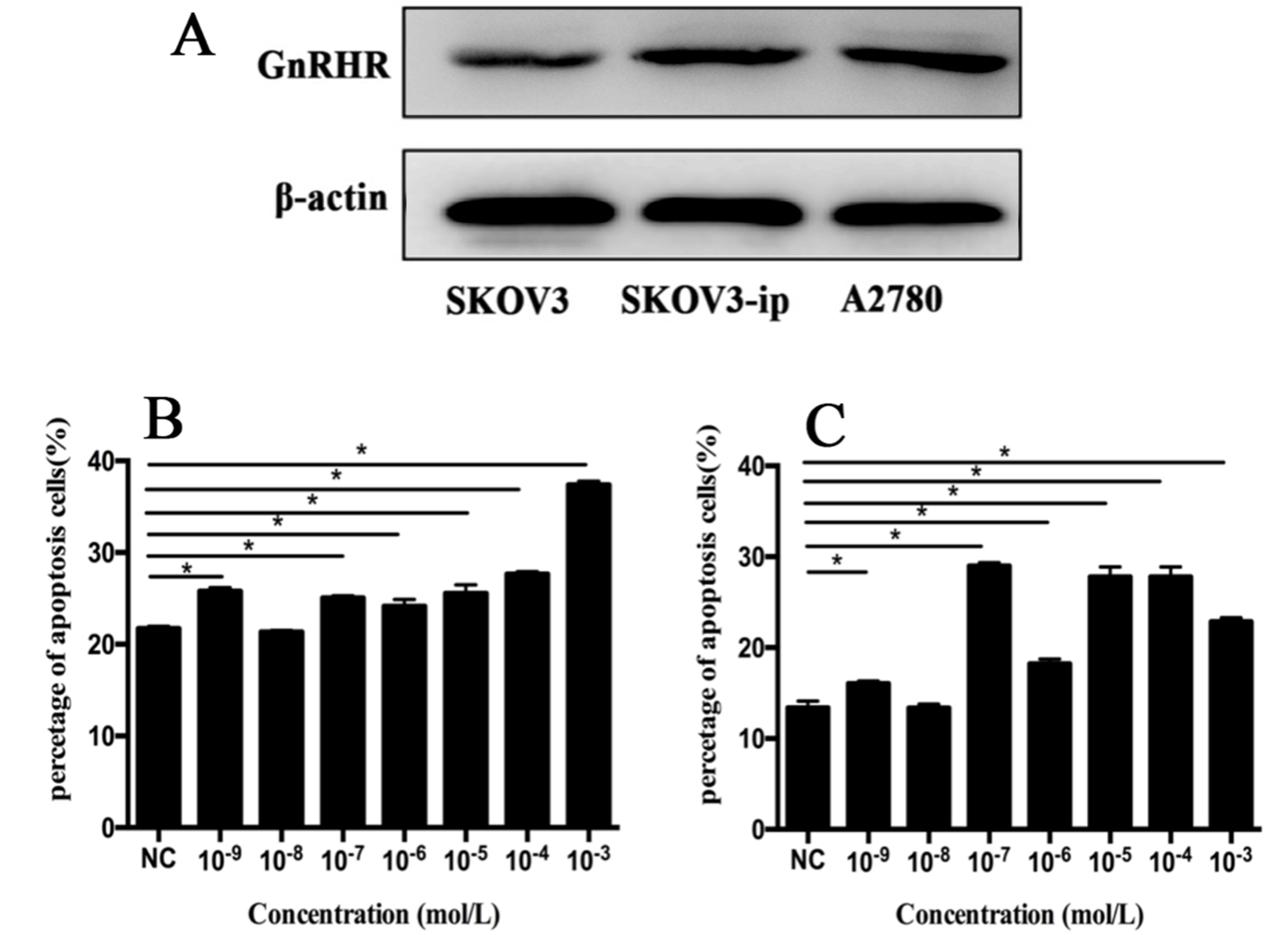


**Supplementary Figure 1.** The expression of GnRHR in EOC cells and the pro-apoptotic effect by different concentrations of cetrorelix at different time-points. (A) Western blot analysis of GnRHR expression in SKOV3, SKOV3-ip and A2780 cells. Flow cytometric analysis of the total apoptosis rate of SKOV3-ip cells treated with cetrorelix for 48h (B) and 72 h (C) (**P*<0.05).


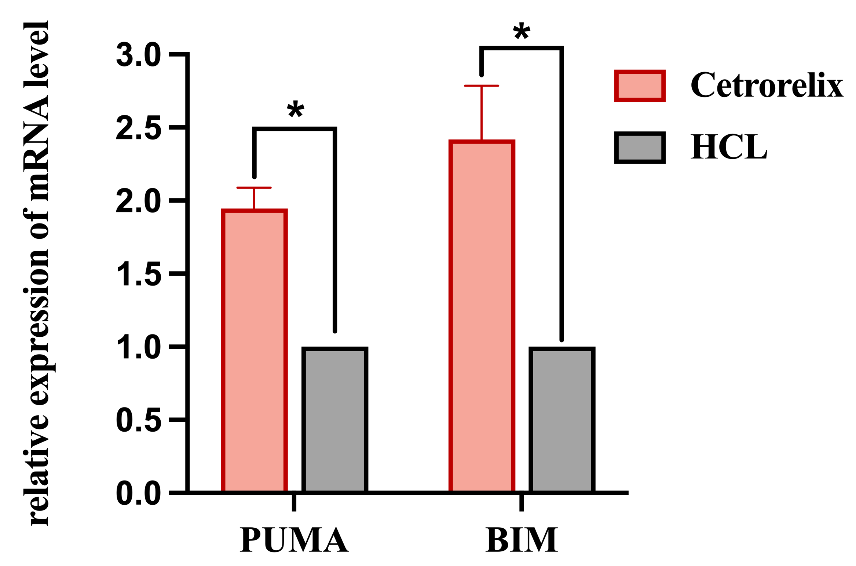
**Supplementary Figure 2.** Cetrorelix upregulates the expression of FOXO1 target genes BIM, and PUMA.The mRNA levels of key FOXO1 transcriptional targets, BIM and PUMA, were significantly increased upon cetrorelix treatment (10^-4^ mol/L, 48 h) in SKOV3 cells, as determined by qRT-PCR. (**P*<0.05).

## Supplementary Tables

**Table S1. Primers used in the study**

| Gene | Primer | Sequences(5’-3’) |
| --- | --- | --- |
| FOXO1 | Forward | CGGGTATGTAACTGAACTTG |
|  | Reverse | GCTGTAGTTGCCTCTTTAAT |
| AKT1 | Forward | CTTCAAGCCCCAGGTCAC |
|  | Reverse | CGCTGTCCACACACTCCAT |
| GAPDH | Forward | ACCACAGTCCATGCCATCAC |
|  | Reverse | CCACCACCCTGTTGCTGTAG |
| BCL2L10 | Forward | GGATTCCGTGCTCTCCGACA |
|  | Reverse | TCGCCCTCCTGCTCCTTTAG |
| CASP14 | Forward | CCACGGTAGAGGGATACATCG |
|  | Reverse | GAGGGTGCTTTGGATTTCAGG |
| CASP5 | Forward | GACTCTCCAGCATCCTTGGC |
|  | Reverse | CTACGTCCCACCCAAGTGAC |
| CIDEA | Forward | GCCGAAGAGGTCGGGAATAG |
|  | Reverse | TTCACGTTAAGGCAGCCGAT |
| FASLG | Forward | CTTCCCTGTCCAACCTCTGTG |
|  | Reverse | CTACCAAGGCAACCAGAACCA |
| TNFSF8 | Forward | TGTTGGTCGTTCAGAGGACG |
|  | Reverse | TGGAGGTAGGCCCATGACTT |
| TNFRSF10A | Forward | GGTCAAGGATTGTACGCCCT |
|  | Reverse | CTGAGCCGATGCAACAACAG |
| TNFRSF9 | Forward | TGTGCTTGTGAATGGGACGA |
|  | Reverse | GAAACGGAGCGTGAGGAAGA |
| BIM | Forward | TCCTCCTTGCCAGGCCTT |
|  | Reverse | CTGCAGGTTCAGCCTGCC |
| PUMA | Forward | GACGACCTCAACGCACAGTA |
|  | Reverse | CACCTAATTGGGCTCCATCT |

| **Table S2. Clinical features of 104** **serous adenocarcinoma patients** | | |
| --- | --- | --- |
| **Variables Numbers(%)** | | |
| *Age(years)* |  |  |
| <50 | 30(28.85%) | |
| ≥50 | 74(71.15%) | |
| *Histological grade* |  |  |
| High grade | 79(75.96%) | |
| Low-grade | 25(24.04%) | |
| *FIGO stage* |  |  |
| I-II | 41(39.42%) | |
| III-IV | 63(60.58%) | |
| *Lymph node metastasis* |  |  |
| Absent | 77(74.04%) | |
| Present | 27(25.96%) | |
| *Distant metastasis* |  |  |
| Absent | 48(46.15%) | |
| Present | 56(53.85%) | |
| *Cancer cell in ascites* |  |  |
| Absent | 68(65.38%) | |
| Present | 36(34.62%) | |
| *Residual tumor diameter(cm)* |  |  |
| <1 | 57(54.80%) | |
| ≥1 | 47(45.19%) | |

**Table S3. Eight differentially expressed genes (fold change ≥1.5) were screened in the SKOV3-ip-Cetrorelix group**

| **Gene Name** | **GeneBank ID** | **Description** | **Fold change** |
| --- | --- | --- | --- |
| BCL2L10 | NM_020396 | BCL2-like 10 (apoptosis facilitator) | -1.85 |
| CASP14 | NM_012114 | Caspase 14, apoptosis-related cysteine peptidase | 2.17 |
| CASP5 | NM_004347 | Caspase 5, apoptosis-related cysteine peptidase | 2.21 |
| CIDEA | NM_001279 | Cell death-inducing DFFA-like effector a | 3.17 |
| FASL | NM_000639 | Fas ligand (TNF superfamily, member 6) | 4.68 |
| TNFRSF9 | NM_001561 | Tumor necrosis factor receptor superfamily, member 9 | 8.62 |
| TNFSF8 | TNFSF8 | Tumor necrosis factor (ligand) superfamily, member 8 | 2.5 |
| TNFRSF10A | TNFRSF10A | Tumor necrosis factor receptor superfamily, member 10a | 4608.2 |
